# A flexible control on electromagnetic behaviors of graphene oligomer by tuning chemical potential

**DOI:** 10.1186/s11671-018-2762-4

**Published:** 2018-11-03

**Authors:** Junbo Ren, Guangqing Wang, Weibin Qiu, Houbo Chen, Pingping Qiu, Qiang Kan, Jiao-Qing Pan

**Affiliations:** 1 Fujian Key Laboratory of Light Propagation and Transformation, College of Information Science and Engineering, Huaqiao University, Xiamen, 361021 China; 2 College of Materials Science and Opto-Electronic Technology, University of Chinese Academy of Sciences, Beijing, 100086 China; 3 Institute of Semiconductors, Chinese Academy of Sciences, Beijing, 100086 China

**Keywords:** Graphene oligomer, Surface plasmon, Electromagnetic tunability, Optical absorption

## Abstract

In this work, we demonstrate that the electromagnetic properties of graphene oligomer can be drastically modified by locally modifications of the chemical potentials. The chemical potential variations of different positions in graphene oligomer have different impacts on both extinction spectra and electromagnetic fields. The flexible tailoring of the localizations of the electromagnetic fields can be achieved by precisely adjusting the chemical potentials of the graphene nanodisks at corresponding positions. The proposed nanostructures in this work lead to the practical applications of graphene-based plasmonic devices such as nanosensing, light trapping and photodetection.

## Introduction

Recently, an increasing number of subwavelength components and structures have been designed and manufactured based on metamaterials (MMs) which get spotlight by the versatility of controlling the electromagnetic (EM) behaviors [1]. MMs support unique phenomena which cannot exist in nature including negative refractive index [2], extraordinary optical transmission [3], and electromagnetically induced transparency [4]. Due to unique properties of MMs, the nanodevices composed of MMs have more prominent advantages that the nanodevices possess pronounced and flexible ability to regulate and control EM behaviors, which leads the development of nanodevices toward high quality and integrability. Plasmonic MMs is one kind of metamaterials that exploits surface plasmons (SPs) to achieve novel optoelectric properties [5, 6]. SPs are the oscillations of free electrons in metal, originating from the interaction of light with metal-dielectric materials. Under certain circumstances, the interaction of incident light with the surface plasmons is able to produce self-sustaining, propagating electromagnetic waves known as surface plasmon polaritons (SPPs) which propagate along the metal-dielectric interface [7]. The SPPs are much shorter than incident light in wavelength, which is suitable for nanostructures with subwavelength footprint [8]. Light hitting the plasmonic MMs is transformed into SPPs, leading to the appearance of strong field localization in these structures at the resonance frequencies. The EM properties of plasmonic structures are primarily controlled by their geometry, making it possible to optimize the electric and magnetic behaviors over a broad range [9–12]. In practice, electron-beam lithography and focused-ion beam milling are two common methods to fabricate plasmonic structures on planar substrates. The excellent EM behaviors stem from the unique features of plasmonic structures with characteristics smaller than the wavelength of light separated by subwavelength distances, revealing a striking way to design applications in nanoscale such as sensing [13], surface-enhanced spectroscopies [14], and nonlinear optics [15]. Most common plasmonic MMs are composed of gold and silver which exhibit negative real permittivity [16]. However, noble metals have relatively large ohmic loss and low flexibility that once the structure is fixed, the EM behaviors cannot be optimized further, which restricts the development of nanodevices based on plasmonic structures [17, 18].

Graphene is one two-dimensional material made up of sp^2^ hybridization of carbon atoms in the honeycomb lattice structure. Due to the surpassing behaviors in electronics as well as photonics of graphene, various research groups investigate graphene with different methods to create plasmonic structures that exhibit lower losses, higher confinement and tunability of the EM fields [19–23]. Graphene is able to accommodate SPPs in large range from terahertz to mid-infrared frequencies [24–26]. Graphene has great potential for improving light-matter interactions in a two-dimensional regime because of SPs with strong light confinement [27]. Graphene oligomers constitute plasmonic molecules (PMs) via interactions between components, where the EM fields with strong field enhancements follow symmetries analogous to the coupling of atoms in chemical molecules [28]. By changing the chemical potential of graphene, the graphene PMs can reach high quality and flexibility [29]. However, there are more adjustable structure parameters such as chemical potential of graphene in different positions for graphene nanostructures to control the EM behaviors. Most of reported graphene nanostructures concentrate on changing chemical potential of the whole structure, which is short of elucidation of the relationship between chemical potential of graphene in different position and the EM behaviors of graphene nanostructure. The proposed graphene nanostructures may stimulate more surpassing EM properties and will affect a wide range of plasmonic applications.

To verify the effect mechanisms of PMs based on graphene, a numerical study on the graphene oligomer consisting of 13 equal-sized graphene nanodisks has been systemically conducted by intentionally varying chemical potential of partial graphene in this work. The graphene oligomer with D_12h_ symmetry is able to sustain two plasmonic modes in calculated range. Further utilization of graphene oligomer relies on the precise control of local chemical potential of graphene. By selectively varying chemical potentials of graphene oligomer, the two innate plasmonic modes are profoundly modulated. Tuning the chemical potential of prominent graphene nanodisks in two plasmonic modes respectively has different influence on two plasmonic modes. The change of chemical potential of intersection part between the two plasmonic modes intensifies both two plasmonic resonances and leads to degeneration of plasmonic modes. In addition, the change of chemical potential of central graphene nanodisk also significantly affects the EM properties of graphene oligomer. The simulated results show that the graphene oligomer possesses high tunability and flexibility, and provides new degrees of freedom for designing plasmonic nanodevices capable of tailoring two-dimensional light confinement.

## Simulated methods and models

In our model, the graphene is treated as one thin film with one atom layer thickness ∆ and modeled by a complex permittivity ε [22].

1 $$ \upvarepsilon =1+\frac{i{\sigma}_g{\eta}_0}{k_0\Delta}, $$

where ∆ = 0.334 nm, *σ*_*g*_ is the complex surface conductivity of graphene, *ŋ*_0_=377 Ω stands for the impendence of the free space, and *k*_0_ = 2*π*/*λ* is the wave number of the light in air. The complex surface conductivity *σ*_*g*_ of graphene monolayer is modeled by Kubo’s formulation, which consists of contributions from both intraband electron-photon scattering *σ*_*intra*_ and interband electron-electron transition *σ*_*inter*_ [30],

2 $$ {\sigma}_g={\sigma}_{intra}+{\sigma}_{inter}, $$

where

3 $$ {\sigma}_{intra}=\frac{2{e}^2{k}_BT}{\pi {\mathrm{\hslash}}^2}\cdot \frac{i}{\omega +i{\tau}^{-1}}\left[\ln \left(2\cosh \left(\frac{\mu_c}{k_BT}\right)\right)\right], $$

4 $$ {\sigma}_{inter}=\frac{e^2}{4\mathrm{\hslash}}\left[\frac{\sinh \left(\frac{\mathrm{\hslash \upomega }}{2{k}_BT}\right)}{\cosh \left(\frac{\mu_c}{k_BT}\right)+\cosh \left(\frac{\mathrm{\hslash \upomega }}{2{k}_BT}\right)}-\frac{i}{2\pi}\ln \frac{{\left(\mathrm{\hslash}\omega +2{\mu}_c\right)}^2}{{\left(\mathrm{\hslash}\omega -2{\mu}_c\right)}^2+{\left(2{k}_BT\right)}^2}\right]. $$

In these equations, e is the charge of an electron, *ℏ* is the reduced Planck constant, k_B_ is the Boltzmann constant, T is the temperature set as 300 K, τ is the momentum relaxation time set as 0.5 ps, ω is the radian frequency, and *μ*_*c*_ is the chemical potential of graphene.

**Fig. 1:**
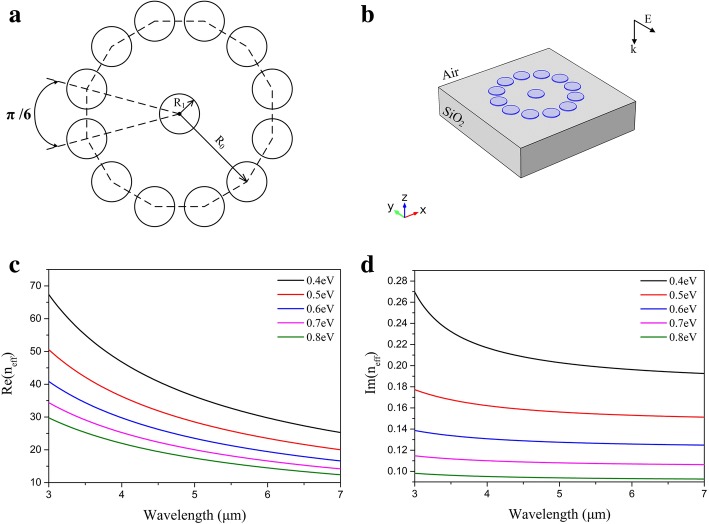
**a** The schematic diagram of the graphene oligomer with symmetry D_12h_ consisting of 13 identical graphene disks. **b** The simulation model of graphene oligomer. The graphene oligomer is placed on the silica substrate with n_2_ = 1.5 and is surrounded by air with n_1_ = 1. **c**, **d** The real part and imaginary part of *n*_eff_ with the chemical potential of graphene ranging from 0.4 to 0.8 eV

We incorporate graphene nanodisk arrays into a graphene oligomer with D_12h_ symmetry (Fig. 1a) to investigate the EM behaviors. The graphene oligomer consists of 13 graphene nanodisks of equal size, where one nanodisk is placed in the center and the others surround it with dodecagon symmetry. The radius of the addendum concentric circle R_0_ is 240 nm and the radius of individual nanodisks R_1_ is 50 nm. The graphene oligomer composed of large number of graphene nanodisks has advantage in flexible selections to change chemical potentials. As shown in Fig. 1b, the graphene oligomer is surrounded with air described by a refractive index n_1_ = 1 and adheres to a silica substrate with a refractive index n_2_ = 1.5. The incident light is vertical to graphene oligomer and the polarization is along y axis. Theoretically, the effective refractive index of graphene is described by

5 $$ {n}_{eff}=\frac{2i{\varepsilon}_{\mathrm{e} ff}{\varepsilon}_0c}{\sigma_g}. $$

where *ε*_eff_ is the effective permittivity of the environment media, *ε*_0_ is the vacuum permittivity and *c* is the speed of light in vacuum. According to the equations (2, 3, 4, and 5), it is seen that *n*_eff_ is a function of *μ*_*c*_ and the relationship is plotted in Fig. 1c and d, meaning that the resonance of our proposed structure can be expediently modified via manipulating the chemical potential of graphene. It should be pointed out that |*Im*(*n*_*eff*_)|/|*Re*(*n*_*eff*_)| is significantly small. So the real part of n_eff_ mainly effect the calculate results and the imaginary part of n_eff_ has little effect on our model with chemical potential changing. We therefore neglect the effect of imaginary part of n_eff_ in this study.

The electric fields and extinction spectra of graphene oligomer are calculated in the commercial finite element method (FEM) software, COMSOL Multi-Physics, RF Module. The extinction cross-section *σ*_*ext*_ is obtained as *σ*_*ext*_ = *σ*_*sc*_ + *σ*_*abs*_, where *σ*_*sc*_ corresponds to the scattering cross-section

6 $$ {\sigma}_{sc}=\frac{1}{I_0}\int \int \left(\overrightarrow{n}\cdot \overrightarrow{S_{sc}}\right) dS, $$

and the absorption cross-section *σ*_*abs*_, is determined by

7 $$ {\sigma}_{abs}=\frac{1}{I_0}\int \int \int \kern0.5em QdV. $$

In these equations, I_0_ is the incident intensity. $\overrightarrow{n}$stands for the normal vector pointing outwards from the plasmonic nanocluster, $\overrightarrow{S_{SC}}$indicates the Poynting vector for the scattered field. The integral in Equation (6) is taken over the closed surface of the scatter. Q is the power loss density in the oligomer. The integral in equation (7) is taken over its volume. The extinction spectra are calculated in the selected wavelength range of mid-infrared. The Perfectly Matched Layer (PML) is applied around the proposed nanostructure to avoid the reflected light fields. The thickness of the graphene is meshed at least five layers to guarantee simulation accuracy.

## Simulation results and discussions

### The effect of local chemical potential change of graphene nanodisks in plasmonic modes

**Fig. 2:**
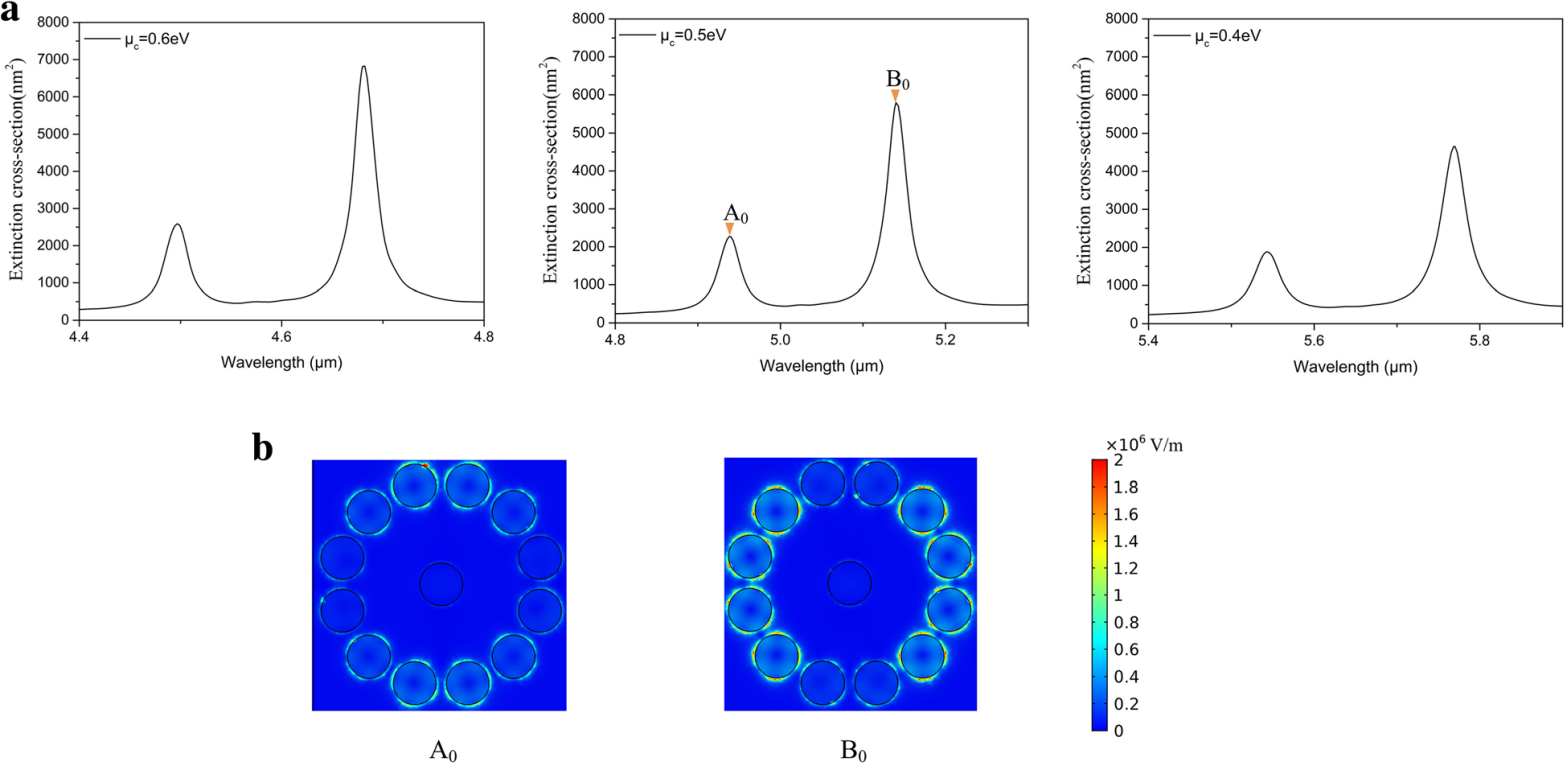
**a** The extinction spectra of graphene oligomer with the chemical potential ranging from 0.4 to 0.6 eV. **b** The simulated electric fields (|E|) at the two resonance peaks

**Fig. 3:**
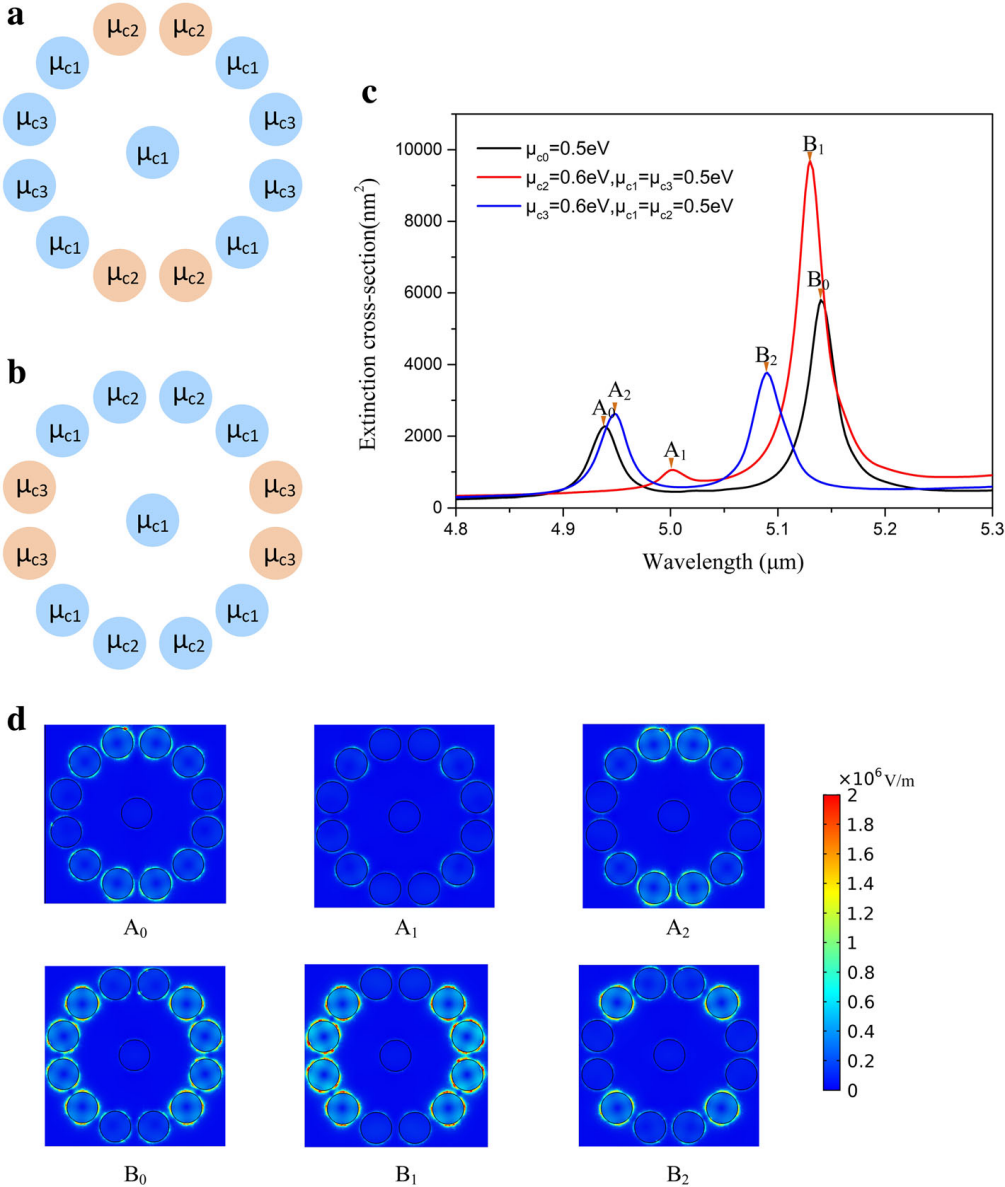
**a**, **b** Schematic illustration of selectional graphene nanodisks with different chemical potential change in graphene oligomer. **c** The extinction spectra with different chemical potentials. **d** The simulated electric field (|E|) at the resonance peaks A_0_, A_1_ and A_2_, B_0_, B_1_ and B_2_

For the proposed structure, the extinction spectra (Fig. 2) exhibit two prominent resonances associated with the excitation of plasmons in the graphene oligomer. The graphene oligomer is able to sustain two plasmonic modes, which are both sensitive to the graphene chemical potential *μ*_*c*_. By varying *μ*_*c*_ of the whole graphene oligomer from 0.4 eV to 0.6 eV, both two plasmonic resonances become intense, and the positions move to higher frequency range simultaneously. The distinct enhancement of absorption in graphene oligomer is ascribed to the promotion of carrier density with increasing *μ*_*c*_, which creates an optical gap where plasmons avoid being quenched through coupling to electron−hole pairs (Landau damping). The increase of virtual electron-hole pair transitions allowed gives rise to the significant interaction of coherently coupled graphene nanodisks which intensifies extinction maximum [21]. We choose the extinction spectrum with *μ*_*c*_ = 0.5eV as the benchmark and the two peaks labeled by A_0_ and B_0_ represent two different plasmonic modes and the corresponding electric fields are presented in Fig. 2b. Strong concentrated electric fields appear as the nanoscale electromagnetic hot spot and lead to extinction enhancement. For peak A_0_, the hot spots mainly concentrate on the eight nanodisks on the top and bottom, and especially focus on the four nanodisks on the highest and lowest positions in the nanostructure. For peak B_0_, the hot spots mainly concentrate on the eight nanodisks on the left side and right side, and the brightest four nanodisks are on the most left and right positions in the nanostructure, which is perpendicular to the mode of peak A_0_. Based on the different electric field distributions of peak A_0_ and B_0_, we define the mode of peak A_0_ as Y mode and the mode of peak B_0_ as X mode for a clear expression. The four brightest graphene nanodisks in Y mode are extremely dark in X mode and vice versa. Another four graphene nanodisks composed of a square are relatively bright both in Y mode and X mode defined as the intersection part. We divide the peripheral graphene nanodisks into three parts with different chemical potentials *μ*_*c*1_, *μ*_*c*2_ and *μ*_*c*3_ respectively (shown in Fig. 3a and b). The nanodisks with *μ*_*c*2_ or *μ*_*c*3_ are the brightest part in Y mode or X mode. The chemical potential of intersection part and center *μ*_*c*1_ keeps 0.5 eV in next calculation. At first, *μ*_*c*2_ increases to 0.6 eV and others keep 0.5 eV (shown in Fig. 3a). Then *μ*_*c*3_ increases to 0.6 eV and others keep 0.5 eV (shown in Fig. 3b). By changing *μ*_*c*2_ or *μ*_*c*3_ to 0.6 eV respectively, a series of spectral variations visibly appear in Fig. 3c. We can see that by changing the chemical potential of sectional graphene nanodisks and leaving the other parameters constant, a flexible reconfiguration of the overall spectral shape is obtained, manifested by a systematic change in the height of two resonance peaks. In Fig. 3d, the electric fields of variant Y mode and X mode are plotted in detail. As shown in Fig. 1c, the real part of n_eff_ is inversely proportional to the chemical potential. Therewith when the chemical potential increases, the confinement of the incidence light becomes weak. The mechanism of local chemical potential change in graphene oligomer is that, the increase of chemical potential reduces the interaction between light and the graphene nanodisks, and pushes the hot spots to surrounding nanodisks. If the orientation of pushing is to the location of strong plasmonic resonance, the resonance is strikingly strengthened, otherwise it is reduced. This means that the effect of local chemical potential change relies on the electric field distributions of different modes. When *μ*_*c*2_ increases to 0.6 eV, peak A_0_ significantly decreases and red shift to peak A_1_ due to the weak confinement of the four brightest graphene nanodisks for the incidence light, where the hot spots mainly concentrate on the intersection part. Simultaneously, peak B_0_ significantly increase and blue shift to peak B_1_, which is attributed to the fact that the increase of *μ*_*c*2_ sufficiently enhance the X mode. For *μ*_*c*3_=0.6 eV, it is the other way round. Peak A_0_ slightly increases and red shifts to peak A_2_ arising from the enhancement of Y mode with *μ*_*c*3_ increasing. In the meantime, peak B_0_ blue shifts to peak B_2_ and decreases with the concentration of hot spots on the intersection part, which is in accord with peak A_1_.

These variations of Y mode and X mode give rise to the descent or enhancement in extinction spectra. A flexible control over the extinction curves is achieved by adjusting the EM behaviors of Y mode and X mode arising from selectively adding the chemical potentials of graphene nanodisks, which opens a novel path way for designing graphene nanodevices with different functions. For example, when *μ*_*c*2_ = 0.6eV, peak A_0_ become lower while peak B_0_ significantly intensifies, which makes the graphene oligomer suitable for high efficient absorbers. In the other way, when *μ*_*c*3_ = 0.6eV, the values of two peaks approach closely, which is convenient to design dual-band nanosensors.

### The mode enhancement by increasing chemical potential of the intersection part

**Fig. 4:**
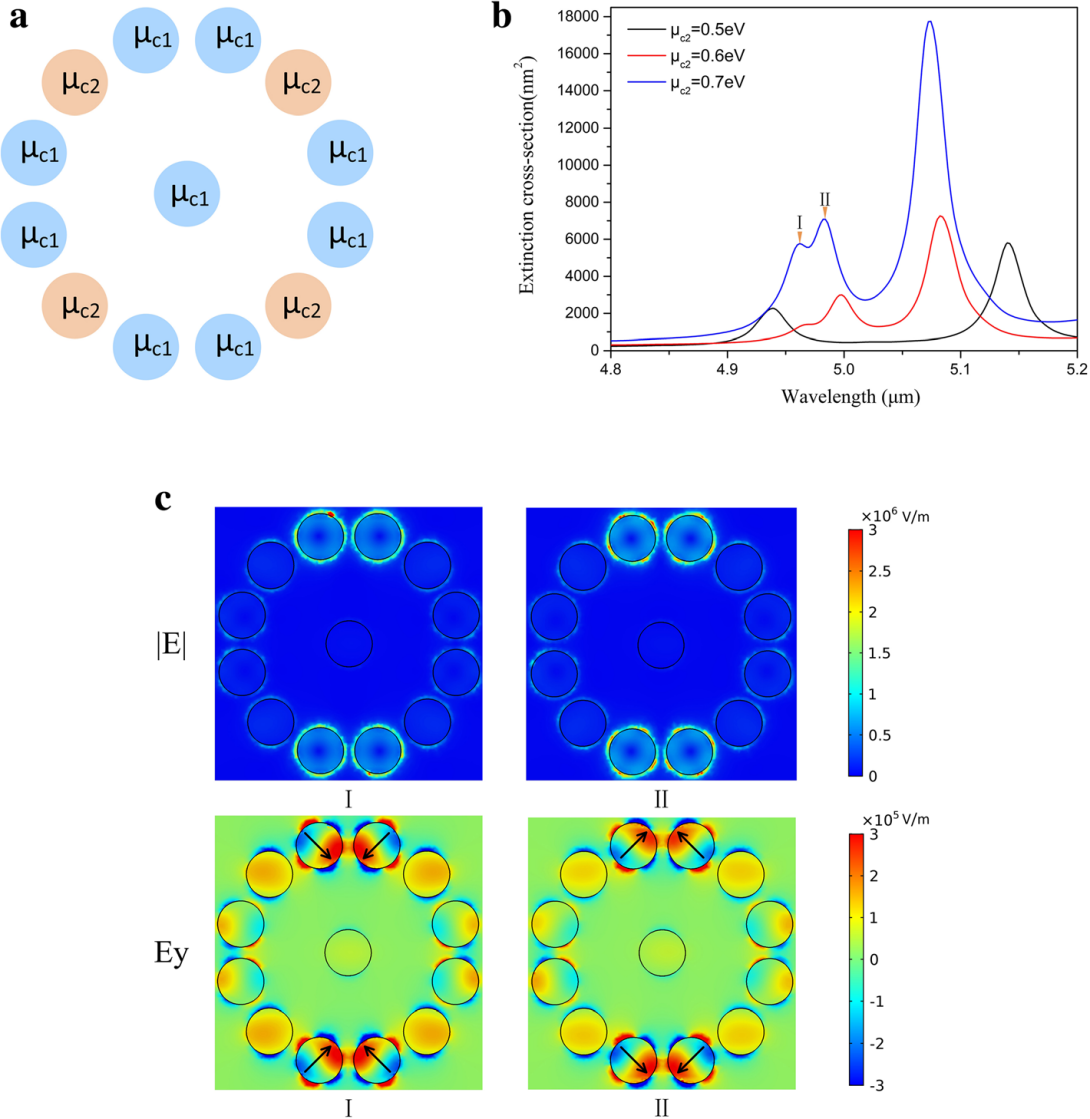
**a** Schematic illustration of selectional graphene nanodisks with different chemical potentials to change chemical potential of intersection part. **b** The extinction spectra with increasing chemical potential of intersection part from 0.5 eV to 0.7 eV. **c** The electric fields (|E|) and the electric fields of y component (Ey) at the resonance peaks I and II

For the electromagnetic fields of two plasmonic modes, an intersection part composed of four graphene nanodisks between two plasmonic modes appears. As shown in Fig. 3d, the electric fields mainly concentrate on the four graphene nanodisks in the intersection part by locally changing the chemical potential. So we believe that the chemical potential of intersection part significantly influences the EM characteristics of graphene oligomer and profile of extinction spectra. We redistribute the chemical potentials in graphene oligomer. The chemical potential of four graphene nanodisks in intersection part is set as *μ*_*c*2_. The chemical potential of other nanodisks *μ*_*c*1_ keeps at 0.5 eV (shown in Fig. 4a). On the basic of the mechanisms of local chemical potential change, the increasing chemical potential of the intersection part intensifies both Y mode and X mode. As shown in Fig. 4b, with increasing *μ*_*c*2_, the extinction spectrum is drastically modified. When *μ*_*c*2_ increases to 0.6 eV, both two resonance peaks have a promotion compared with *μ*_*c*2_=0.5 eV. It is noted that the there is a new resonance peak appears around the resonance peak of Y mode. When the *μ*_*c*2_ further increases to 0.7 eV, the two resonance peaks become stronger and a new resonance peak obviously appears around the resonance peak of Y mode. The elucidation of strong enhancement of resonance peaks is that the increase of *μ*_*c*2_ efficiently intensifies the both Y mode and X mode. The increase of *μ*_*c*2_ facilitates the plasmonic oscillations of four graphene nanodisk in Y mode and X mode respectively. The resonance peak of Y mode splitting into two resonance peaks is a process of degeneration. As shown in Fig. 4c, the two resonance peaks labeled by I and II have same electric fields but the components of electric field are different. The directions of Ey of peak I and II are perpendicular to each other, which represent two plasmonic modes degenerating from Y mode. The two new plasmonic modes originally merge in Y mode, and the two modes begin to separate with *μ*_*c*2_ increasing. In addition, both two degenerate resonance peaks with *μ*_*c*2_ = 0.6eV are much larger than resonance peak with *μ*_*c*2_ = 0.5eV. In such a manner, by choosing the graphene nanodisks of intersection part to increase their chemical potential, one can improve all resonance peaks in extinction spectra. It is proposed to enhance the absorption of graphene nanodisks by selectively changing the chemical potentials of adaptive graphene nanodisks, which helps to design plasmonic nanodevices capable of light absorption with high efficiency.

### The effect of chemical potential of central nanodisk

**Fig. 5:**
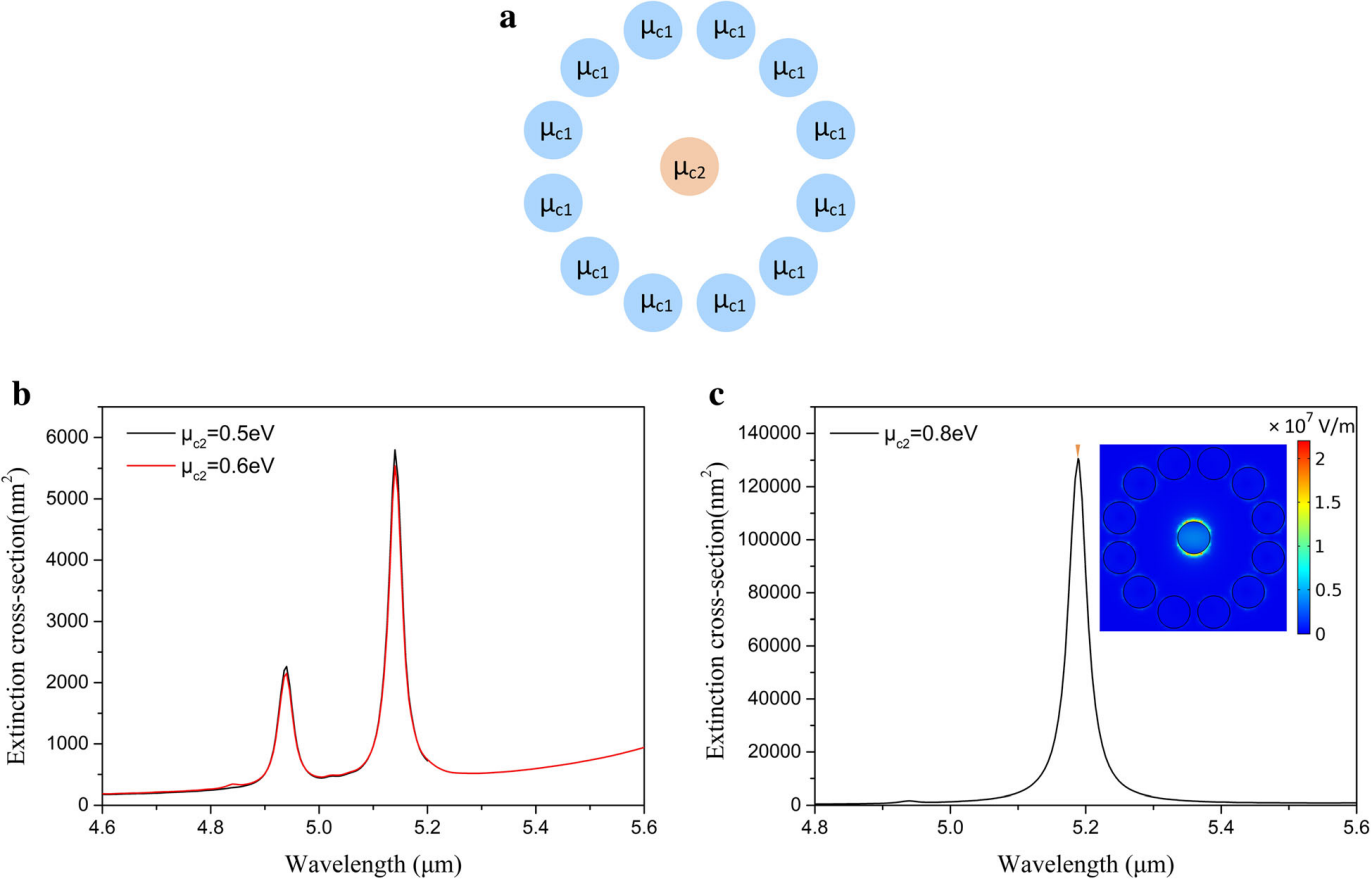
**a** Schematic illustration of selectional graphene nanodisks with different chemical potentials to change chemical potential of central graphene nanodisk. **b** The extinction spectra of graphene oligomer with the chemical potential of central graphene nanodisk *μ*_*c*2_ = 0.5eV and *μ*_*c*2_ = 0.6eV. **c** The extinction spectrum of graphene oligomer with the chemical potential of central graphene nanodisk *μ*_*c*2_ = 0.8eV. The inset shows the electric fields (|E|) at the resonance peak

The central graphene nanodisks introduced into the graphene oligomer is aimed to enable the nanostructures possess more flexibility and further investigate the effect of local chemical change in different positions. Due to the large distance between the central graphene nanodisk and the peripheral graphene nanodisks, the central graphene nanodisk cannot couple with the other graphene nanodisks in two plasmonic modes. In this section, we set the chemical potential of central graphene nanodisk as *μ*_*c*2_. Others are set as *μ*_*c*1_ keeping 0.5 eV (shown in Fig. 5a). Changing the chemical potential of central graphene nanodisk *μ*_*c*2_ is able to modify EM fields of the graphene oligomer without changing the geometry. The results by increasing *μ*_*c*2_ are shown in Fig. 5b and c. The increase of *μ*_*c*2_ enhances the plasmonic oscillations of central graphene nanodisks. However, when increase of *μ*_*c*2_ is relative small, the oscillator strength of central graphene nanodisk is not enough to support new plasmonic mode and influence the intrinsic modes, so the extinction spectrum *μ*_*c*2_ = 0.6eV has almost no change compared with *μ*_*c*2_ = 0.5eV, where two resonance peaks still appear (shown in Fig. 5b). When *μ*_*c*2_ reaches to a large value (0.8 eV), a new resonance peak appears obviously in the extinction spectrum (shown in Fig. 5c). The huge improvement of plasmonic oscillations profoundly changes the profile of extinction spectrum. The new resonance peak originates from the strong interaction between the incident light and the central graphene nanodisk, of which the EM fields mainly concentrate on the central graphene nanodisk, which is defined as the central mode. The resonance peak supported by the central mode is much larger than two intrinsic resonance peaks, while the two intrinsic resonance peaks are drastically suppressed and even disappear in the extinction spectrum. The effect of *μ*_*c*2_ is different from the effect discussed earlier, because the central graphene nanodisk is not contained in the innate plasmonic modes. The effect of *μ*_*c*2_ consists with changing the chemical potential of whole graphene oligomer which is discussed in the beginning. In such a manner, by increasing *μ*_*c*2_, one can design the novel plasmonic device capable of absorbing incident light efficiently. Combining with aforementioned studies, the flexible tailor of the localizations of the electromagnetic field can be achieved by precisely adjusting the chemical potentials of the graphene nanodisk in different positions.

In practice, continuous atomic single-layer of graphene is first grown using an optimized chemical vapor deposition method with CH_4_ as the carbon source. Then graphene film is determined to be monolayer by Raman measurements. The electron-beam lithography with poly(methyl methacrylate) (PMMA) as an electron beam resist is used to pattern the graphene film to produce the proposed nanostructures, and the exposed area is etched away by the oxygen plasma, remaining the pattern of graphene protected by a PMMA layer with subsequent lift-off using acetone. Then the device is ready for test. The chemical potential can be tuned via manipulating chemical and electrostatic doping. For chemical doping, local chemical potential change can be realized by exposing the required graphene nanodisks to HNO_3_ vapor and simultaneously preventing the contact between other graphene nanodisks and HNO_3_ vapor. For electrostatic doping, an appropriate top gate configuration may locally manipulate the chemical potential of graphene by supplying top gate voltage.

## Conclusions

In conclusion, we have demonstrated the versatility of the graphene oligomer to modify the EM behaviors and spectral lineshape by varying chemical potential of graphene at the nanoscale. The characteristics are summarized from the electric fields and extinction spectra of the various chemical potentials. First, by changing the chemical potential of two graphene nanodisks in Y mode and X mode respectively, a flexible variation of two resonance peaks appears in extinction spectra. The two resonance peaks can be enhanced or reduced by changing the different chemical potentials of graphene oligomer. Second, increasing the chemical potential of intersection part intensifies the both two resonance peaks and gives rise to degeneration of Y mode. Third, high chemical potential of central graphene nanodisk is able to support a strong resonance peak and simultaneously restrain two innate resonance peaks. The dependence of the graphene oligomer on chemical potential suggests that one can modify the EM behaviors of the graphene nanostructure with chemical potential without modifying the geometry. The previous studies based on graphene nanostructures can only change one absorption peak by changing chemical potential of whole graphene [19–23], but the method of changing chemical potential of graphene in this paper can tune the spectra with additional flexibility, which brings out more surpassing EM phenomena. In the realm of practical applications, our studies provide a new degree of freedom for modifying the graphene plasmonics by tuning the chemical potential of the graphene nanostructures. The graphene nanostructures provide a facile platform to cultivate the EM behaviors with light in two dimensions, which pave a way for the design of graphene-based plasmonic nanodevices for nanosensing, light trapping and photodetection.

## Abbreviations

EM: Electromagnetic
MMs: Metamaterials
PML: Perfectly Matched Layer
PMs: Plasmonic molecules
SPPs: Surface plasmon polaritons
SPs: Surface plasmons
